# Repair Effects of *Chlorella vulgaris* Extract in Non-Chronic Skin Wounds During the Remodeling Phase

**DOI:** 10.3390/cimb48101067

**Published:** 2026-10-15

**Authors:** Hai-Yao Miao, Qiao He, Xiao Ling, Li Li, You-Jie He, Miao-Miao Guo

**Affiliations:** 1 Beijing Key Laboratory of Plant Resources Research and Development, College of Light Industry Science and Engineering, Beijing Technology & Business University, Beijing 102488, China; 2 Beijing Lan Divine Technology Co., Ltd., Beijing 102200, China; 3 College of Biomass Science and Engineering, Sichuan University, Chengdu 610065, China

**Keywords:** *Chlorella vulgaris* extract, HaCaT, repair effect, TGF-β1/mTOR, cosmeceuticals

## Abstract

Algal polysaccharides have been linked to repair signaling pathways related to transforming growth factor-β1 (TGF-β1), a mediator of skin wound healing. However, to our knowledge, no study has directly confirmed the efficacy of *Chlorella vulgaris* extract in repairing non-chronic wounds, and the underlying mechanism remains unexplored. Hence, we investigated the potential involvement of the TGF-β1/protein kinase B (AKT)/mechanistic target of rapamycin (mTOR) pathway in this process. Following preliminary structural characterization of the *Chlorella vulgaris* extract (CE), its cell migration, proliferation and scratch wound closure rate were determined in a wound remodeling phase HaCaT cell model. Compared to the model group, CE effectively was associated with enhanced cell migration and proliferation. At 100 μg/mL, CE was associated with significant upregulation of aquaporin-3 and filaggrin expression. It was associated with inhibited the nuclear translocation of nuclear factor of activated T-cells 1 and nuclear factor erythroid 2-related factor 2. Mechanistically, CE was associated with increased TGF-β1 secretion and mTOR phosphorylation without significantly altering AKT phosphorylation, suggesting a potential involvement of the TGF-β1/mTOR signaling pathway rather than the canonical AKT/mTOR axis. Hemolysis and chicken chorioallantoic membrane assays indicated satisfactory biocompatibility, supporting the safety of the extract for topical application. These findings suggest a possible association between microalgal extracts and insights for developing natural cosmetic ingredients. However, further studies are required to confirm the mechanistic relationships suggested by these findings.

## 1. Introduction

The skin serves as the body’s primary barrier against the external environment and is frequently subjected to injuries from pollutants, microorganisms, ultraviolet radiation, skin diseases such as atopic dermatitis, and physical or iatrogenic procedures including laser treatments and microneedling [1,2,3]. These non-cheonic wounds close naturally within an expected timeframe; however, the rate of closure, severity of inflammation, and quality of barrier restoration collectively influence the reconstruction of skin structure and function. Consequently, safe, highly effective, and mechanistically well-characterized natural repair compounds are urgently needed.

Skin wound healing proceeds through hemostasis, inflammation, proliferation, re-epithelialization, and barrier restoration. Upon injury, platelet aggregation and fibrin clot formation release platelet-derived factors—including platelet-derived growth factor, vascular endothelial growth factor, and transforming growth factor-β1 (TGF-β1), which recruit immune cells and initiate repair [4,5]. Among these, TGF-β1 plays a central role in regulating fibroblast differentiation, keratinocyte proliferation, and epidermal remodeling [6,7]. During the proliferative phase, keratinocytes migrate and proliferate to re-epithelialize the wound, while fibroblasts deposit extracellular matrix (ECM) and differentiate into myofibroblasts to promote contraction [8,9]. Barrier restoration depends on the terminal differentiation of keratinocytes, during which filaggrin (FLG) and other barrier proteins are sequentially exoressed to form the stratum corneum [10,11].

Multiple signaling pathways govern keratinocyte migration, differentiation, and barrier reconstruction including the nuclear factor of activated T-cells (NFAT) and PI3K/AKT/Nrf2 pathways [12,13,14,15]. Of particular relevance, the TGF-β1/AKT/mechanistic target of rapamycin (mTOR) axis directly regulates keratinocyte behavior and epidermal remodeling: TGF-β1 activates AKT via PI3K, leading to mTORC1/2 phosphorylation [16,17,18]. Elevated phosphorylated protein kinase B (p-AKT) at injury site indicates its involvement in repair response [19]. Ozone oil enhances fibroblast migration and epithelial–mesenchymal transition via PI3K/AKT/mTOR signaling [20], and *Parthenium* flower extract promotes HaCaT cell migration by increasing AKT phosphorylation [21], suggesting that this pathway is a key mechanism linking exogenous active ingredients to improved in keratinocyte function.

Algae are rich in structurally diverse bioactive polysaccharides with documented anti-melanogenesis, antioxidant, anti-inflammatory, anti-aging, and UV-damage repair activities [22,23]. Algae have attracted considerable attention in wound dressings owing to their moisturizing and wound-healing properties. An aqueous extract of *Spirulina obtusapicis* promoted cell migration and wound closure in a previous study [24]. *Laminaria* polysaccharides have demonstrated superior wound contraction relative to 1% *Centella asiatica* in full-thickness excision models, while increasing TGF-β1-responsive immune cells and enhancing antioxidant enzyme activity [25]. Sulfated polysaccharides from *Sargassum* inhibit nitric oxide, prostaglandin E2, and pro-inflammatory cytokine production in lipopolysaccharide (LPS)-stimulated macrophages [26]. Notably, kelp polysaccharides increase TGF-β1-responsive immune cells, suggesting a link between algal polysaccharides and TGF-β1-related repair signaling [27].

However, existing studies have largely focused on macroalgae such as brown and red algae, while systematic research on microalgae—particularly *Chlorella vulgaris*—in skin wound healing remains limited. Recent investigations have begun to explore the wound-repair potential of *Chlorella*. A multifunctional conductive hydrogel (QPMP) based on quaternized chitosan, oxidized pullulan, polypyrrole@polydopamine, and C. vulgaris was shown to continuously produce oxygen under illumination to alleviate wound hypoxia, while also providing antibacterial, antioxidant, and wound exudate/temperature monitoring capacities; in diabetic chronic wound models, it effectively controlled inflammation and promoted collagen deposition, angiogenesis, and wound closure [28]. Another study developed a nutrient matrix-supplying hydrogel loaded with live C. vulgaris (HA-gel@CV), which maintained microalgal viability for more than 7 days through components such as hyaluronic acid, ceramide, and urea, promoted cell proliferation, migration, and angiogenesis in vitro, and attenuated inflammation while improving vascular and tissue regeneration in diabetic wounds in vivo [29]. In addition, a self-assembled hydrogel based on Chlorella extract, gelatin, and polyethylene glycol (CPGel) integrated a “scavenging–remodeling–repair” strategy, exhibiting antibacterial, 65% ROS-scavenging, and immunomodulatory activities; it achieved 100% wound closure within 21 days in diabetic mice and promoted 9-fold angiogenesis and 2-fold type I collagen deposition [30]. C. vulgaris extract has also been demonstrated to stimulate collagen production and suppress UVB-induced MMP-1 expression, thereby preserving extracellular matrix integrity [31].

The bioactive constituents of C. vulgaris include polysaccharides, proteins, peptides, pigments (chlorophylls, carotenoids, and lutein), and antioxidants [32]. Among these, Chlorella Growth Factor (CGF), a complex mixture of nucleic acids, amino acids, peptides, vitamins, and polysaccharides, has been associated with cellular regeneration, enhanced skin cell proliferation, and increased inflammatory cell recruitment, thereby supporting wound healing [33]. Chlorella-derived peptides suppress UVB-induced MMP-1 expression and reduce collagen degradation by downregulating CYR61, AP-1, and MCP-1 signaling [31]. Amino acids derived from Chlorella improve water retention, minimize transepidermal water loss, and sustain cellular hydration [31].

Despite these advances, existing studies have prioritized phenotypic effects such as antioxidant and anti-inflammatory properties, and in-depth analyses of the molecular mechanisms regulating specific signaling pathways (such as AKT/mTOR) are lacking. Although the TGF-β1/AKT/mTOR pathway is closely associated with skin cell migration, epithelial–mesenchymal transition, and epidermal homeostasis [19,20,21,22,23,24], and some algal polysaccharides can modulate TGF-β1-related responses [28,30], no study has directly confirmed the efficacy of *Chlorella vulgaris* in repairing non-chronic wounds or its association with this pathway.

This study systematically evaluated the effects of *Chlorella vulgaris* extract (CE) on non-chronic skin wound healing and investigated the regulatory mechanisms of the TGF-β1/AKT/mTOR pathway in this process using an in vitro HaCaT model to assess cell migration, proliferation, and barrier protein expression.

## 2. Materials and Methods

### 2.1. Reagents and Equipment

The reagents procured were as follows: *Chlorella vulgaris* (Xi’an Tongze Biotechnology Co., Ltd., Xi’an, China); human immortalized keratinocytes (HaCaT, Cell Bank of the China Type Culture Collection, Shanghai, China); fresh sheep blood (Beijing Ji Wei Biotechnology Co., Ltd., Beijing, China); specific-pathogen-free chicken embryos (White Leghorn) (Beijing Boehringer Ingelheim Viton Biotechnology Co., Ltd., Beijing, China); Dulbecco’s Modified Eagle Medium (DMEM), fetal bovine serum, penicillin–streptomycin solution, trypsin, cell cryopreservation medium, and PBS, pH = 7.4 (Bioagro Technology Co., Ltd., Beijing, China); CCK-8 Kit, LPS, DAPI nuclear stain, and DMSO (Beijing Bairuiji Biotechnology Co., Ltd., Beijing, China); 4% paraformaldehyde solution, 30% Triton X-100, bovine serum albumin (BSA), TGF-β1 ELISA Kit, horseradish peroxidase-labeled anti-rabbit IgG (H+L), horseradish peroxidase-labeled anti-mouse IgG (H+L), pre-stained protein marker (10–250 kDa), Bicinchoninic Acid Protein Concentration Assay Kit, Total Protein Extraction Kit, 10× Tris-Buffered Saline with Tween^®^ 20, Enhanced Chemiluminescence (ECL) High-Sensitivity Chemiluminescence Kit, and FITC-labeled goat anti-rabbit IgG (H+L) (Shanghai Biyuntian Biotechnology Co., Ltd., Shanghai, China); anti-Nrf2 antibody and anti-AQP3 antibody (Abcam, Waltham, MA, USA); anti-NFAT1 antibody, phospho-AKT antibody, AKT antibody, and β-actin antibody (Cell Signaling Technology, Danvers, MA, USA); anti-FLG antibody (Affinity, Cincinnati, OH, USA); TGX™ FastCast™ Acrylamide Kit, Blotting Kit, and 10× Tris/Glycine/SDS (Bio-Rad, Hercules, CA, USA); 5× SDS-PAGE loading buffer (Beijing Kangwei Century Biotechnology Co., Ltd., Beijing, China).

The following equipment were used in the study: 1/100,000 electronic balance (Mettler Toledo, Greifensee, Switzerland); HERAcell 150i CO_2_ incubator (Thermo Fisher Scientific, Waltham, MA, USA); microplate reader (TECAN GmbH, Männedorf, Switzerland); benchtop centrifuge (Eppendorf, Hamburg, Germany); super-resolution laser-scanning confocal microscope (Nikon Seiki (Shanghai) Co., Ltd., Shanghai, China); freeze dryer (VirTis, Gardiner, NY, USA); electric-heated high-pressure steam autoclave (Shanghai Boxun Medical Biological Instrument Corp., Shanghai, China); laminar flow hood (Beijing Shiankelin Purification Technology Co., Ltd., Beijing, China); CO_2_ incubator (Thermo Fisher Scientific (China) Co., Ltd., Shanghai, China); chemiluminescence image analysis system (Shanghai Tianneng Technology Co., Ltd., Shanghai, China); rapid blotting system (Bio-Rad, Hercules, CA, USA); UV spectrophotometer (Thermo Fisher Scientific, Waltham, MA, USA); FTIR spectrometer (PerkinElmer, Waltham, MA, USA); 18-angle laser light scatterometer and differential detector (Wyatt, Santa Barbara, CA, USA); electric heating constant-temperature water bath (Beijing Chang’an Scientific Instrument Factory, Beijing, China); inverted microscope (Olympus (China) Co., Ltd., Beijing, China); Waters e2695 Separations Module (Waters Corporation, Milford, MA, USA); Shodex SUGAR KS-805 and KS-803 GPC columns (Resonac Corporation, Tokyo, Japan).

### 2.2. Experimental Methods

#### 2.2.1. Extraction of CE

After grinding the dried algal bodies using a grinder, they were passed through a 100-mesh sieve, and the powder was collected and stored in an airtight container. Varying amounts of the powdered sample were weighed, and a corresponding volume of deionized water was added at a solid-to-liquid ratio of 1:25; the solution was heated in a 90 °C water bath for 2 h for extraction. The mixture was then filtered and concentrated using a rotary evaporator until the supernatant volume was reduced to 100 mL or less. Subsequently, ethanol precipitation was performed by adding at least four times the volume of anhydrous ethanol, followed by placement into a 4 °C refrigerator for 12 h. The sample was centrifuged at 1700× *g* for 10 min; the ethanol precipitate was collected and transferred to a Petri dish, dried in a drying oven, and ground to a powder in a mortar, to obtain the CE.

#### 2.2.2. Determination of the Polysaccharide Content in the CE

A 2.0 mg/mL glucose standard solution was prepared and diluted proportionally to 0.1, 0.2, 0.3, 0.4, 0.5, 0.6, 0.7, and 0.8 mg/mL. To 1.0 mL of each of these standard solutions, 0.5 mL 5% phenol solution was added and mixed thoroughly, immediately followed by the addition of 2.5 mL of concentrated sulfuric acid to each solution and mixing. The mixture was placed in a boiling water bath for 30 min for hydrolysis. Subsequently, the samples were placed in a cold-water bath to cool to 25–28 °C. Next, 200 μL of the reaction solution was placed in a 96-well plate; distilled water was used as the blank control instead of the standard solution. The absorbance was measured at OD490 using a microplate reader, a standard curve was generated based on the experimental data, and the corresponding linear regression equation was determined. Further, a 1.0 mg/mL solution of CE was prepared. Using the same procedure, the OD_490_ of the polysaccharide solution was determined, and the polysaccharide content was determined based on the standard curve.

#### 2.2.3. Preliminary Structural Characterization of the CE

UV Full-Scan Spectroscopy: A 5.0 mg/mL solution of CE was prepared, with distilled water serving as the blank control. The solution was scanned over the wavelength range of 190–700 nm; the UV absorption spectrum was plotted with wavelength on the x-axis and absorbance on the y-axis. Attenuated total reflection Fourier-transform infrared (ATR-FTIR) spectroscopy was employed to identify the major functional groups and anomeric configurations in the CE. A small amount of the lyophilized polysaccharide powder was placed directly onto the ATR diamond crystal and firmly pressed with a built-in anvil to ensure optimal contact. The spectra were recorded over the wavenumber range of 4000–400 cm^−1^ at a resolution of 4.00 cm^−1^, with a scan speed of 0.20 cm/s (mirror velocity). The background spectrum was collected under identical conditions prior to sample measurement. GPC molecular weight analysis: A 3.0 mg/mL solution of the algal polysaccharide was prepared and filtered through a 0.45 µm aqueous filtration membrane. The molecular weight of the CE was determined using an 18-angle laser light scattering instrument coupled with a differential refractive index detector. The following experimental conditions were used: liquid chromatography system, Waters e2695 system; GPC columns, a combination of Shodex SUGAR KS-805 and KS-803 (Resonac Corporation, Tokyo, Japan); injection volume, 0.1 mL; flow rate, 0.8 mL/min; column temperature, 60 °C; mobile phase, a mixture of 0.1 mol/L sodium nitrate solution and 0.02% sodium azide solution.

#### 2.2.4. Establishment of a HaCaT Cell Model for the Remodeling Phase of Non-Chronic Wounds

Non-cytotoxic LPS concentrations were determined using the CCK-8 assay. Here, 2.5 mg/mL LPS was diluted with serum-free DMEM to concentrations of 40, 20, 10, 5, and 2.5 μg/mL. Cells were cultured in Dulbecco’s modified Eagle’s medium supplemented with 10% fetal bovine serum and 1% penicillin–streptomycin at 37 °C in a humidified atmosphere with 5% CO_2_. HaCaT cells at passage 3 were used for all subsequent experiments. The HaCaT cells were seeded at a density of 1 × 10^4^ cells per well in a 96-well plate by adding 100 µL of the cell suspension to each well. The plates were incubated in a constant-temperature, constant-humidity CO_2_ incubator for 24 h until the cells were fully adherent. The 96-well plate was then rinsed with PBS to remove residual complete medium, and 100 µL of each LPS concentration was added. Serum-free DMEM was used as the blank control; three replicate wells were set for each concentration, and the plates were incubated for 24 h. After incubation, the 96-well plate was removed and further processed in the dark. To each well, 10 µL of CCK-8 was added, and the plates were placed in a constant-temperature, constant-humidity CO_2_ incubator for 1 h. A microplate reader was used to measure the absorbance at 450 nm, and the cell survival rate was calculated as follows:

survival rate (%) = (OD of treated sample − OD of blank)/(OD of control − OD of blank) × 100(1)

A non-chronic wound cell model was established using a scratch assay combined with LPS induction. Horizontal lines were drawn on the back of a 48-well plate using a marker pen, with five evenly spaced, parallel lines crossing each well. HaCaT cells were seeded at a density of 6 × 10^4^ cells per well in a 48-well plate by adding 500 µL of cell suspension to each well. The plates were incubated in a constant-temperature, constant-humidity CO_2_ incubator for 24 h until the cells were fully adherent. A 200 µL pipette tip was used to mark scratches perpendicular to the horizontal lines marked previously. The plates were rinsed with PBS to remove residual complete medium; 500 µL of LPS was then added at various concentrations, using 1% DMEM as the blank control. The cells were incubated in a constant-temperature, constant-humidity CO_2_ incubator. At the 0 and 24 h time points, the cells were visualized microscopically and imaged.

#### 2.2.5. Assessment of the Effects of CE on Barrier-Related Proteins Using Immunofluorescence

The sterile slides were soaked in PBS (to keep them moist) and then placed in a 24-well plate pre-marked with a marker pen. HaCaT cells were seeded at a density of 3 × 10^5^ cells per well in a 24-well plate. Then, 500 µL of the cell suspension was added to each well, and the plates incubated in a constant-temperature, constant-humidity CO_2_ incubator for 24 h until the cells were fully adherent. Using a 200 µL pipette tip, a horizontal line was drawn across the well. The supernatant was discarded from the sample wells and washed three times with PBS. Next, 16 µL of 2.5 mg/mL LPS and 500 µL CE (100 μg/mL) diluted in 1% DMEM; the blank control used was 1% DMEM. The 24-well plate was placed in a constant-temperature, constant-humidity CO_2_ incubator and incubated for 24 h before harvesting.

After the cell supernatant was aspirated, the cells were washed three times with PBS. The cells were fixed by adding 100 µL of 4% paraformaldehyde solution to each well, and the plates were incubated at 25–28 °C for 15 min. The wells were washed thoroughly three times with PBS and incubated for 5 min after each wash. Further, 100 µL of 0.3% Triton X-100 diluted in PBS was added to each well, and cell permeabilization was performed for 10 min. The wells were washed thoroughly with PBS three times and incubated for 5 min after each wash. Further, 100 µL 5% BSA diluted in PBS was added to each well, and the plates were incubated at room temperature for 2 h. Without further washing, 100 µL of primary antibody (diluted in 5% BSA in PBS)—FLG (1:200) and AQP3 (1:200)—was added to each well and incubated at 4 °C for 12 h. The wells were thoroughly washed three times with PBS and incubated for 5 min after each wash. Subsequently, the fluorescent secondary antibody was prepared in the dark. To each well, 100 µL of the fluorescent secondary antibody (diluted 1:500 in 5% BSA in PBS) was added in the dark, and the plates were incubated at room temperature for 1.5 h. Further, the wells were washed thoroughly with PBS three times and incubated for 5 min after each wash. To each well, 100 µL of DAPI stain was added in the dark and incubated at room temperature for 10 min. The wells were washed thoroughly with PBS three times and incubated for 5 min after each wash. Using tweezers, the slides were removed from the slide holder and placed on a microscope slide; photographs were captured using a fluorescence microscope.

#### 2.2.6. Western Blot Analysis to Determine the Effects of CE on the AKT/mTOR Pathway

The concentrations of CE used in this study were determined based on our preliminary cell viability/proliferation assay. In brief, cells were treated with different concentrations of CE, and the results showed that cell viability was below 100% at 100 μg/mL, whereas it was above 100% at 200 μg/mL and 400 μg/mL, with 400 μg/mL showing a proliferative effect. Based on these findings, 50, 100, and 200 μg/mL were selected for the subsequent experiments examining TGF-β1 secretion and the AKT/mTOR signaling pathway. The rationale for this concentration range was to include a lower concentration (50 μg/mL), an intermediate concentration corresponding to the viability threshold (100 μg/mL), and a higher concentration with a proliferative effect (200 μg/mL), thereby allowing the dose-dependent effects of CE to be evaluated under conditions that maintained appropriate cell viability. The 400 μg/mL concentration was not used in the signaling pathway experiments because 200 μg/mL already represented a concentration with enhanced cell viability, and the selected range was sufficient to assess dose-dependent regulation of TGF-β1 and AKT/mTOR.

Cells were seeded as described in Section 2.2.5. The cells were harvested after reagent application and incubation for 24 h. Proteins were then extracted according to the instructions for the total protein extraction kit. The extracted proteins were denatured, and 20–30 µg of each protein sample was loaded; the loading volume was calculated based on the actual concentration of each sample. The loading mixture was prepared on ice in a volume ratio of 4:1 (protein sample or protein marker: 5× loading buffer). The metal bath was preheated to 100 °C; the protein sample mixture was placed in the bath and heated for 5 min to complete denaturation. Proteins were separated by sodium dodecyl sulfate–polyacrylamide gel electrophoresis (SDS-PAGE) using a 12% (for proteins < 30 kDa) or 10% (for proteins > 30 kDa) separating gel with a 5% stacking gel at a constant voltage of 100 V. Following electrophoresis, the separated proteins were transferred onto polyvinylidene difluoride (PVDF) membranes using the Bio-Rad Trans-Blot^®^ Turbo™ Transfer System with the pre-programmed “Turbo” protocol for mini gels (25 V constant, 1.3 A, 7 min). The proteins were then blocked with 5% BSA for 2 h and incubated with the primary antibody for 12 h at 4 °C. Subsequently, incubation was performed with the secondary antibody. Protein bands were visualized using an enhanced chemiluminescence (ECL) substrate. The membranes were incubated with the ECL working solution for 3 min at room temperature in the dark, and the chemiluminescent signals were captured using a gel imaging system. The exposure time was automatically determined by the imaging system to ensure optimal signal intensity without saturation. Densitometric analysis of the bands was performed using ImageJ1 software. All experiments were independently repeated at least three times (*n* = 3 biological replicates), and representative blots are shown.

#### 2.2.7. Assessment of the Effect of CE on TGF-β1 Levels Using ELISA

The 1 mg/mL CE was diluted to 200, 100, and 50 μg/mL using 1% DMEM. HaCaT cells were seeded at a density of 1 × 10^6^ cells/mL into a six-well plate by adding 2 mL of cell suspension to each well. The plates were incubated in a constant-temperature, constant-humidity CO_2_ incubator for 24 h until the cells were fully adherent. A 200 μL pipette tip was used to draw a horizontal line perpendicular to the markings on the plate. The plates were rinsed with PBS to remove residual complete medium. Subsequently, 16 µL of 2.5 mg/mL LPS and 2 mL of each CE dilute solution was added, using 1% DMEM as the blank control. The six-well plates were placed in a constant-temperature, constant-humidity CO_2_ incubator and incubated for 24 h. The changes in TGF-β1 levels in the test sample wells were determined according to the instructions of the TGF-β1 ELISA Kit. All the reagents were incubated at room temperature for 30 min before use.

#### 2.2.8. Erythrocyte Hemolysis Test

To separate 1.5 mL centrifuge tubes, 500 μL of varying CE at various concentrations (diluted in PBS) was added, along with an equal volume of a 2% (*w*/*w*) sheep red blood cell suspension. The tubes were mixed thoroughly, and three replicates for each sample were prepared using PBS as the negative control and 1% DMSO solution as the positive control. The centrifuge tubes were incubated in a 37 °C constant-temperature incubator for 1 h and subsequently centrifuged at 900 × g for 5 min. From each sample, 200 μL of the supernatant was transferred to a 96-well plate. With PBS as the blank control, the absorbance was measured at 540 nm using a microplate reader. The hemolysis rate was calculated subsequently.

HR (%) = [(A_sample − A_negative)/(A_positive − A_negative)] × 100%(2)

where A_sample, A_negative, and A_positive represent the absorbance values of the sample, negative control, and positive control, respectively.

#### 2.2.9. CAM Safety Test

In this experiment, fertilized chicken eggs (SPF White Leghorn) were incubated at 37.8 °C and 60% relative humidity in a humidified incubator, with the eggs placed horizontally and turned every 2 h until they reached embryonic day 9 (E9), at which point the embryos were used for experiments. The blank, positive control, and treatment groups each consisted of five chicken embryos. The test liquid was applied to the chorioallantoic membrane (CAM) using a pipette, and timing was initiated once the test liquid slightly covered the CAM surface. Membrane reactions, including hemorrhage, vascular lysis, and coagulation, were observed and recorded within 5 min. According to the Irritation Score (IS) method, the time (in seconds) for the onset of each reaction was recorded, and the IS was calculated using the following formula:

(3) $$\mathrm{IS}=\frac{\left(301-\sec\;H\right)\times 5}{300}+\frac{\left(301-\sec\;L\right)\times 7}{300}+\frac{\left(301-\sec\;C\right)\times 9}{300}$$

Based on the calculated IS values, the irritation potential of the test sample was classified as follows: non-irritating (IS < 1), mildly irritating (1 ≤ IS < 5), moderately irritating (5 ≤ IS < 9), or severely irritating/corrosive (IS ≥ 10). An IS between 9 and 10 is considered a borderline zone requiring further confirmation. All experiments were independently repeated at least three times, and data were expressed as mean ± SD.

Here, sec H (hemorrhage time) represents the average time (in seconds) until bleeding is observed; sec L (vessel lysis time) represents the average time (in seconds) until vascular lysis is observed; and sec C (coagulation time) represents the average time (in seconds) until coagulation is observed.

#### 2.2.10. Data Processing and Analyses

All experiments were independently repeated at least three times (*n* = 3 independent biological replicates). Statistical analyses were performed using SPSS Statistics (version 26.0, IBM Corp., Armonk, NY, USA). Data were expressed as mean ± standard deviation (SD). For comparisons among multiple groups, one-way analysis of variance (ANOVA) was employed, followed by Tukey’s post hoc test for multiple comparisons. A significance level of α = 0.05 was set, and *p*-values less than 0.05 were considered statistically significant. Immunofluorescence and Western blot band densities were quantified using ImageJ1 software (National Institutes of Health, Bethesda, MD, USA). Graphical representations were generated using GraphPad Prism (version 8.0, GraphPad Software, San Diego, CA, USA).

## 3. Results

### 3.1. Determination of Polysaccharide Content and Preliminary Structural Characterization of the CE

**Figure 1:**
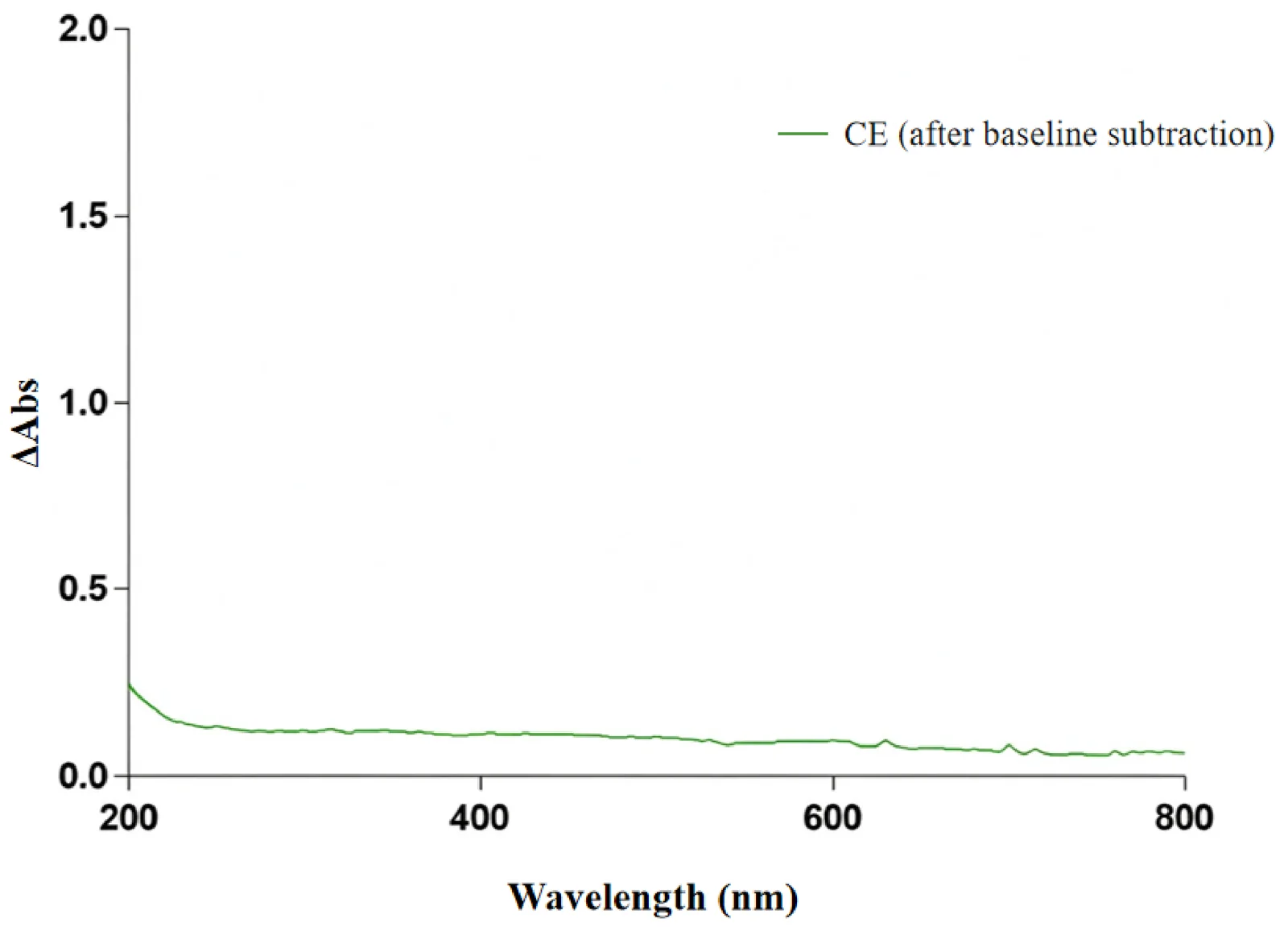
UV-Visible full-scan spectrum of the CE.

The total sugar content in the alcohol-precipitated CE was quantitatively analyzed using the phenol–sulfuric acid method; a standard curve was established using glucose as the reference. The total sugar content in the extract was 11.48 ± 0.25% (based on the dry weight of the extract, *n* = 3). To evaluate the presence of biomacromolecular impurities in the extract, a full-wavelength UV spectrum scan was performed (Figure 1). The extract exhibited no distinct characteristic absorption peaks at 260 nm (for nucleic acids) or 280 nm (for proteins), indicating low absorbance background in the corresponding wavelength ranges.

**Figure 2:**
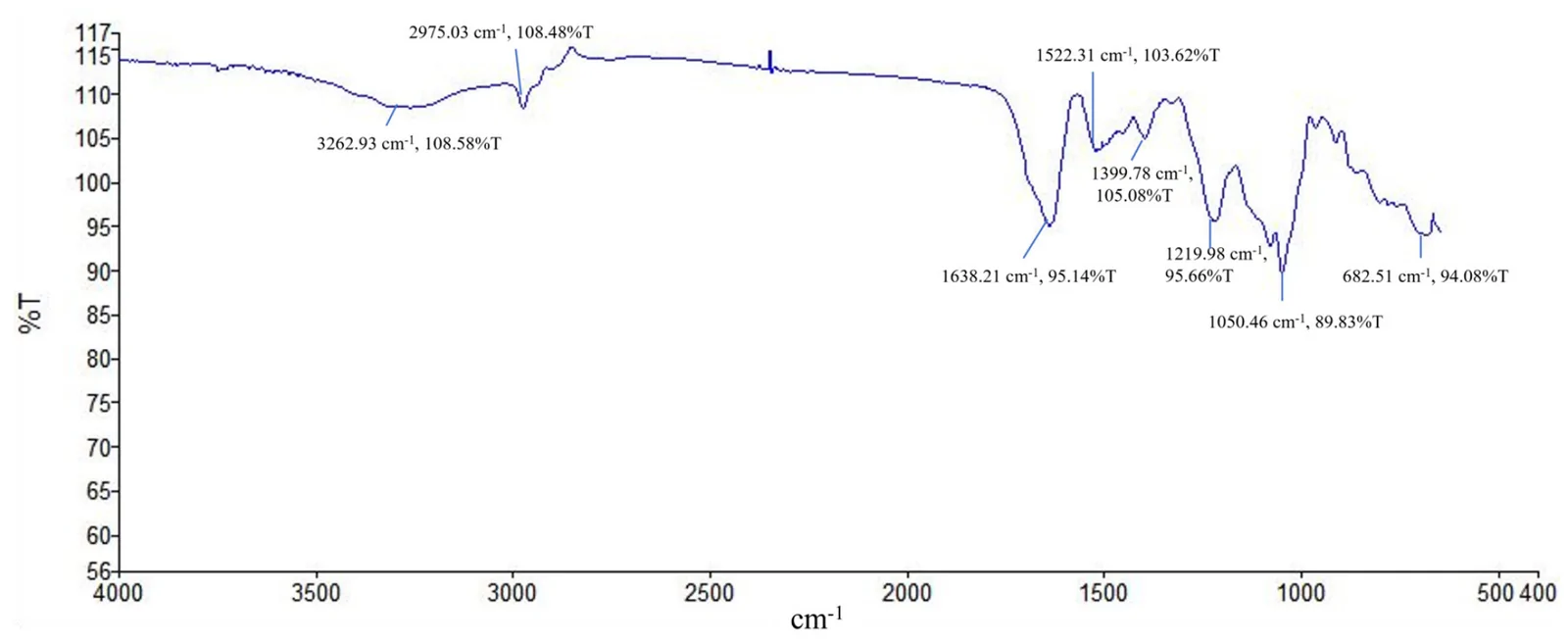
Fourier-transform infrared spectroscopy (FTIR) spectra of the CE.

An infrared (IR) spectrometer was used to characterize the functional group structure of the extract in the range of 4000–400 cm^−1^ (Figure 2, Table 1). The IR absorption spectrum of the extract was consistent with the typical spectral characteristics of polysaccharide compounds. A broad absorption feature was observed in the 3200–3400 cm^−1^ region, attributed to the stretching vibration of hydroxyl (–OH) groups. The absorption peak near 2975 cm^−1^ is attributed to the stretching vibration of the C–H bond on the sugar ring. Two distinct absorption peaks were observed at 1638 cm^−1^ and 1399 cm^−1^, corresponding to the asymmetric and symmetric stretching vibrations of the carboxylate ion (COO^−^), respectively; indicating the presence of uronic acid residues. Within the characteristic fingerprint region for sugars (1200–1000 cm^−1^), absorption peaks from the C–O–C and C–O–H stretching vibrations were detected at 1219 cm^−1^ and 1050 cm^−1^, respectively, with the strong, broad peak at 1050 cm^−1^ being characteristic of the furanose ring skeleton.

**Figure 3:**
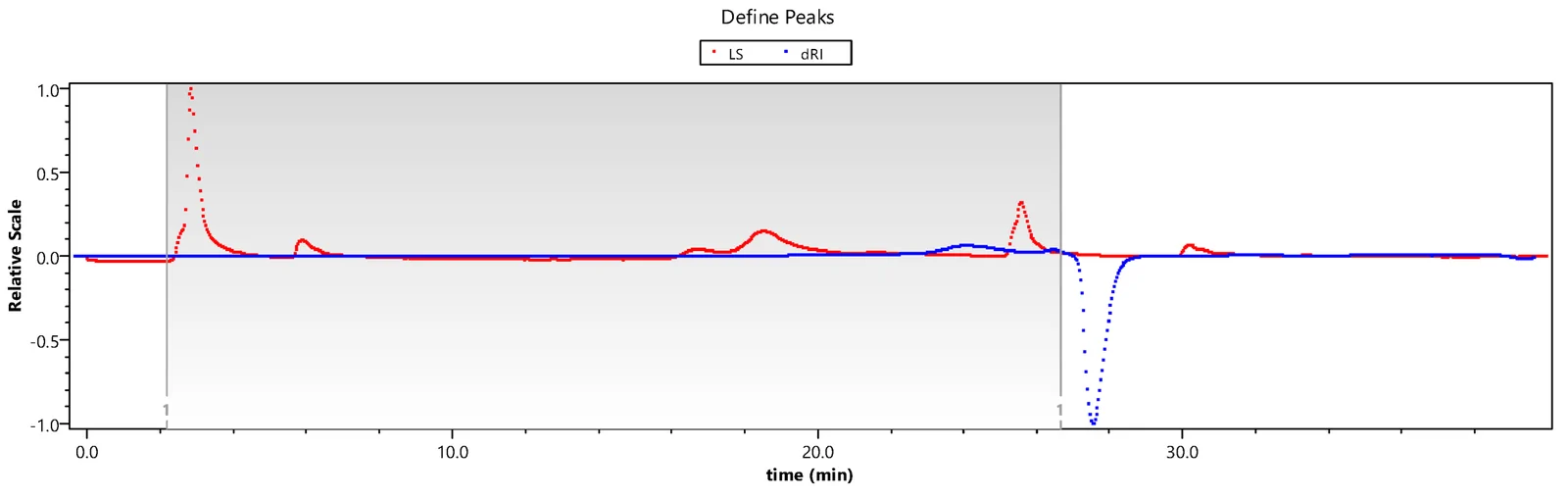
Gel permeation chromatography (GPC) profile of the CE.

Gel permeation chromatography (GPC) was used to determine the molecular weight and molecular weight distribution of the extract (Figure 3). The resulting chromatogram revealed a single, but broad distribution peak. Based on the calibration curve of the standard, the number-average molecular weight (Mn), weight-average molecular weight (Mw), and peak molecular weight (Mp) of this sample were calculated as 1.61 × 10^7^ Da, 1.07 × 10^8^ Da, and 1.18 × 10^7^ Da, respectively.

The polydispersity index (PDI = Mw/Mn) calculated from the above data was 6.65, which is significantly greater than 1.0, further confirming that molecules with varying sizes constituted the extract.

### 3.2. Establishment of a HaCaT Cell Model for Non-Chronic Wounds Induced by a Combination of Cell Scratch Assay and LPS

**Figure 4:**
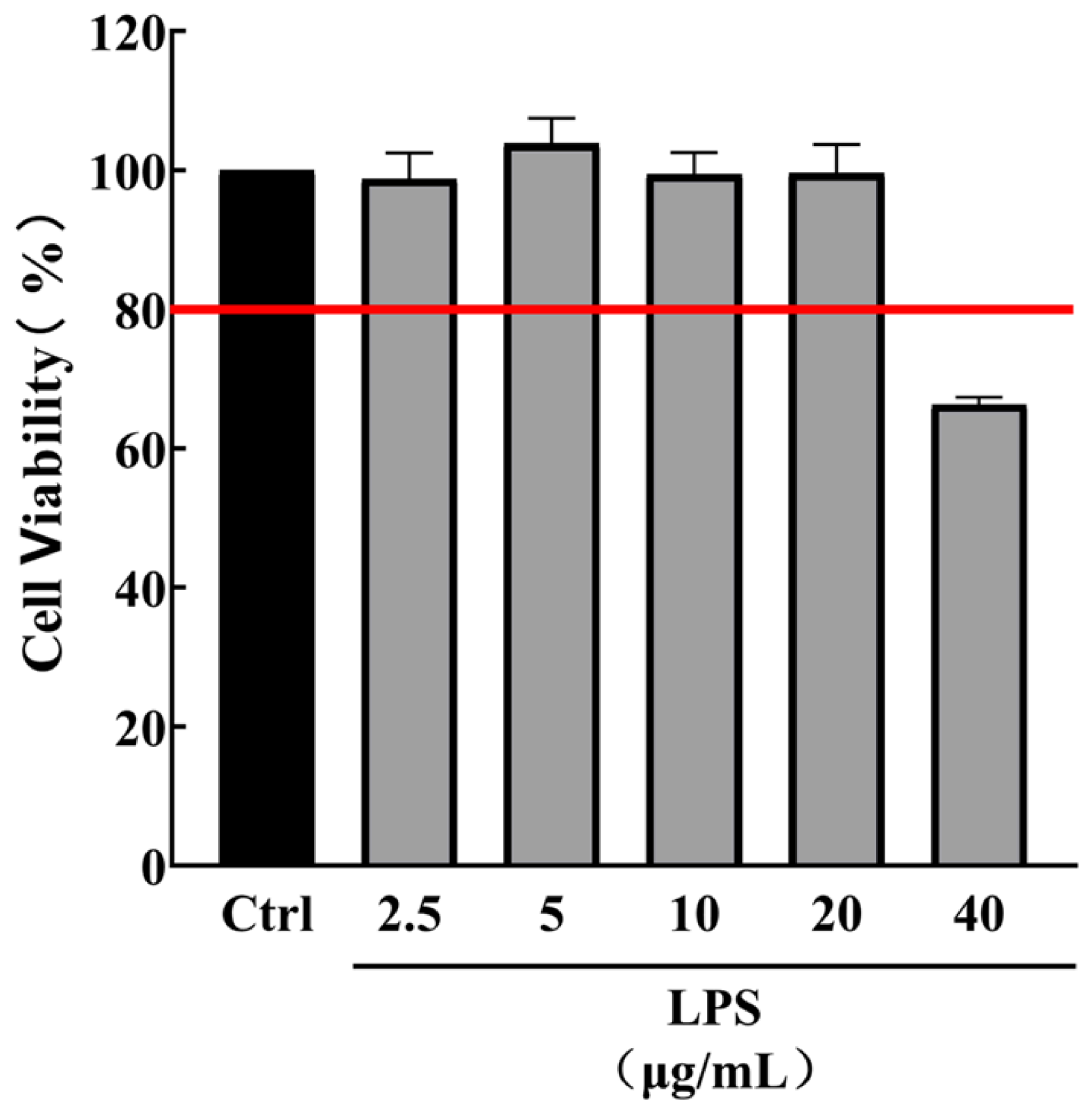
Viability of HaCaT cells treated with various LPS concentrations. The red line representing an 80% cell survival rate.

The Cell Counting Kit-8 (CCK-8) assay was used to assess the effects of different LPS concentrations (0, 2.5, 5, 10, 20, and 40 μg/mL) on the survival rate of HaCaT cells. Within the concentration range of 0–20 μg/mL, the cell survival rates of all LPS-treated groups were approximately 100%, with no significant difference relative to the blank control group; when the LPS concentration was increased to 40 μg/mL, the cell survival rate dropped below 80% (Figure 4).

**Figure 5:**
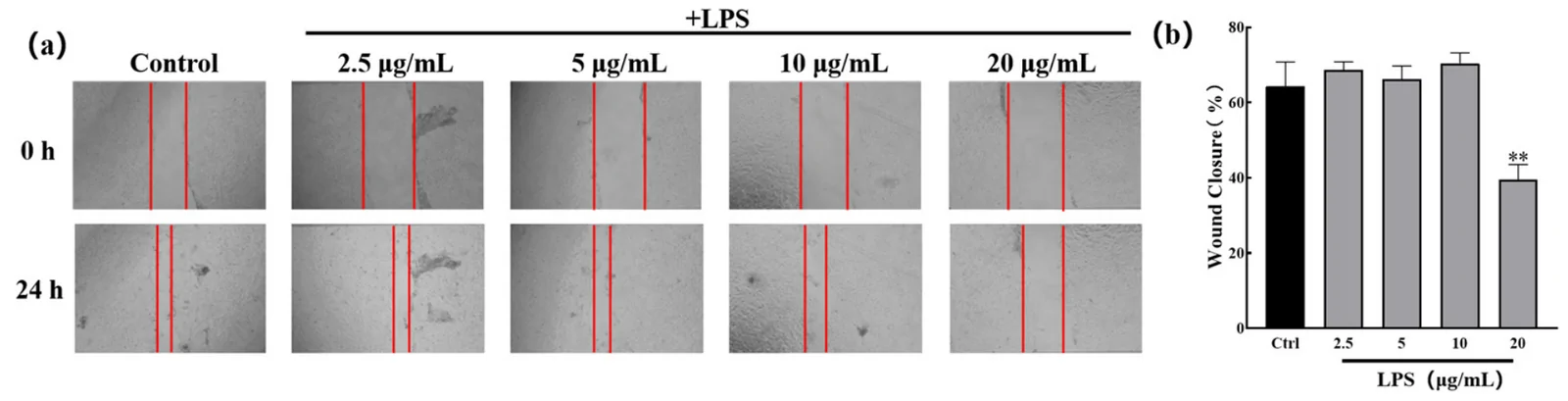
Effect of various LPS concentrations on the migration of HaCaT cells. (**a**) Microscopic images showing the effect of different LPS concentrations on the migration of HaCaT cells (0 and 24 h). (**b**) Quantitative results demonstrating the effect of different LPS concentrations on the migration rate of HaCaT cells. ** *p* < 0.01 vs. Control group.

The effects of different LPS concentrations (0, 2.5, 5, 10, and 20 μg/mL) on the migratory capacity of HaCaT cells were evaluated using a scratch assay. Images were captured at 0 and 24 h after the scratch was made (Figure 5a), and the scratch area was measured using ImageJ1 software to calculate the migration rate (Figure 5b). Compared with the migration rate of the blank control group, those of the 2.5, 5, and 10 μg/mL LPS-treated groups showed an upward trend; in contrast, the migration rate in the 20 μg/mL LPS-treated group was significantly reduced, reaching only 39.27% (*p* < 0.01). Furthermore, the CCK-8 assay results revealed that the cell survival rate in the 20 μg/mL LPS-treated group remained above 80%.

Migration rate = (Initial area − area after 24 h)/Initial area × 100%(4)

### 3.3. Effects of the CE on HaCaT Cell Viability and Its Impact on Cell Migration in a Cell Model

**Figure 6:**
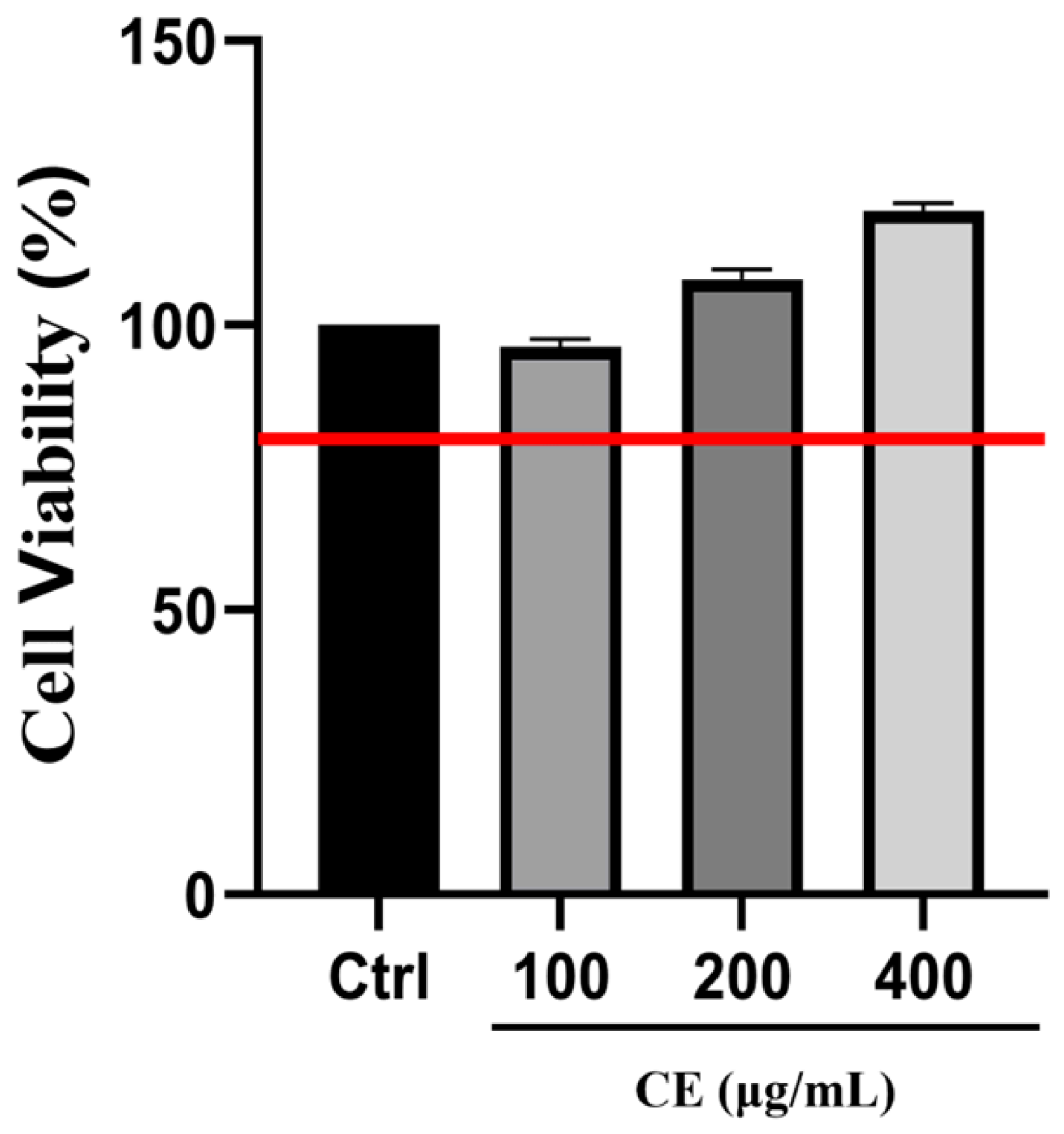
Viability of HaCaT cells treated with various concentrations of the CE. The red line representing an 80% cell survival rate.

The CCK-8 assay was used to evaluate the cytotoxicity of CE at different concentrations (0, 100, 200, and 400 μg/mL) after treating the HaCaT cells for 24 h. The cell survival rate in all treatment groups was higher than 80%, and no significant decline was observed across the different concentration groups (Figure 6).

**Figure 7:**
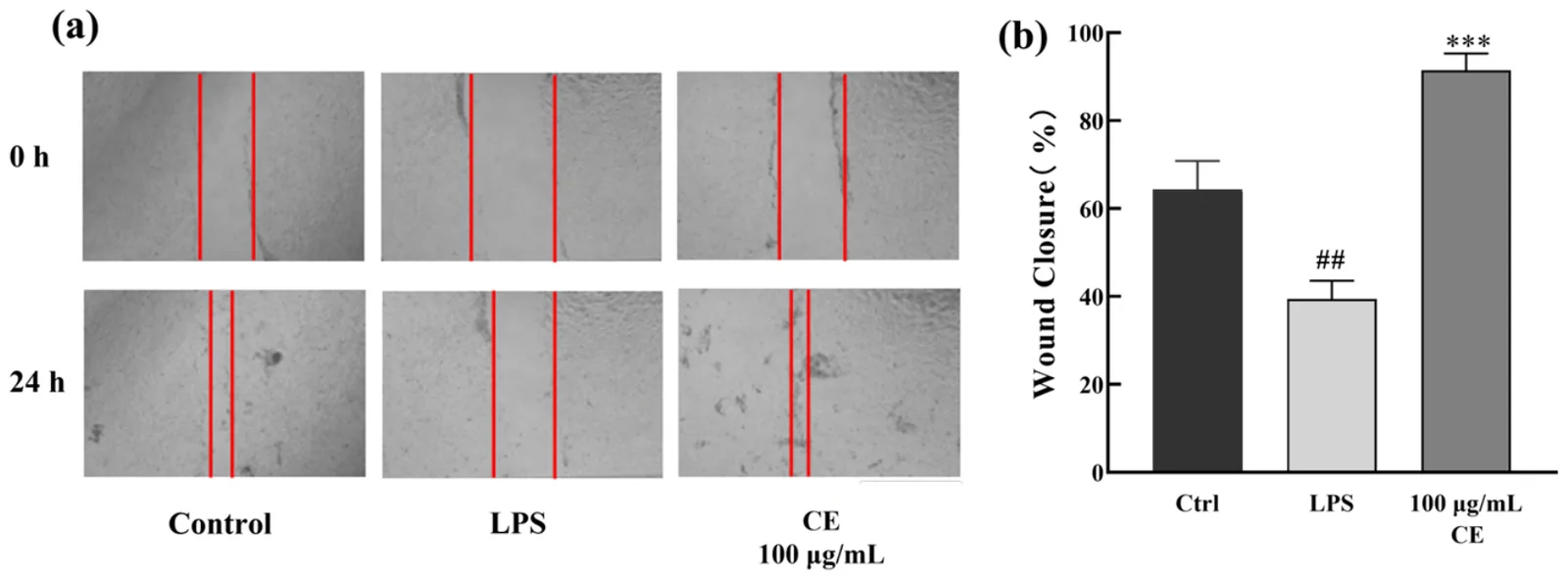
Effect of the CE on the migration rate in an in vitro wound healing model. (**a**) Microscopic images showing the effect of the CE on the migration of HaCaT cells (0 h, 24 h). (**b**) Quantitative results showing the effect of the CE on the migration rate of HaCaT cells. ## *p* < 0.01 vs. C group; *** *p* < 0.001 vs. LPS group.

The effect of the CE on HaCaT cell migration was assessed using a scratch assay. Images were captured at 0 h and 24 h after the scratch was made (Figure 7a), and the scratch area was measured using the ImageJ1 software to calculate the migration rate (Figure 7b). The results revealed that the cell migration rate significantly increased to 91.42% (*p* < 0.01) after 24 h of treatment with 100 μg/mL CE, compared with that of the control group.

### 3.4. Effects of the CE on the Expression of Barrier Repair Proteins

**Figure 8:**
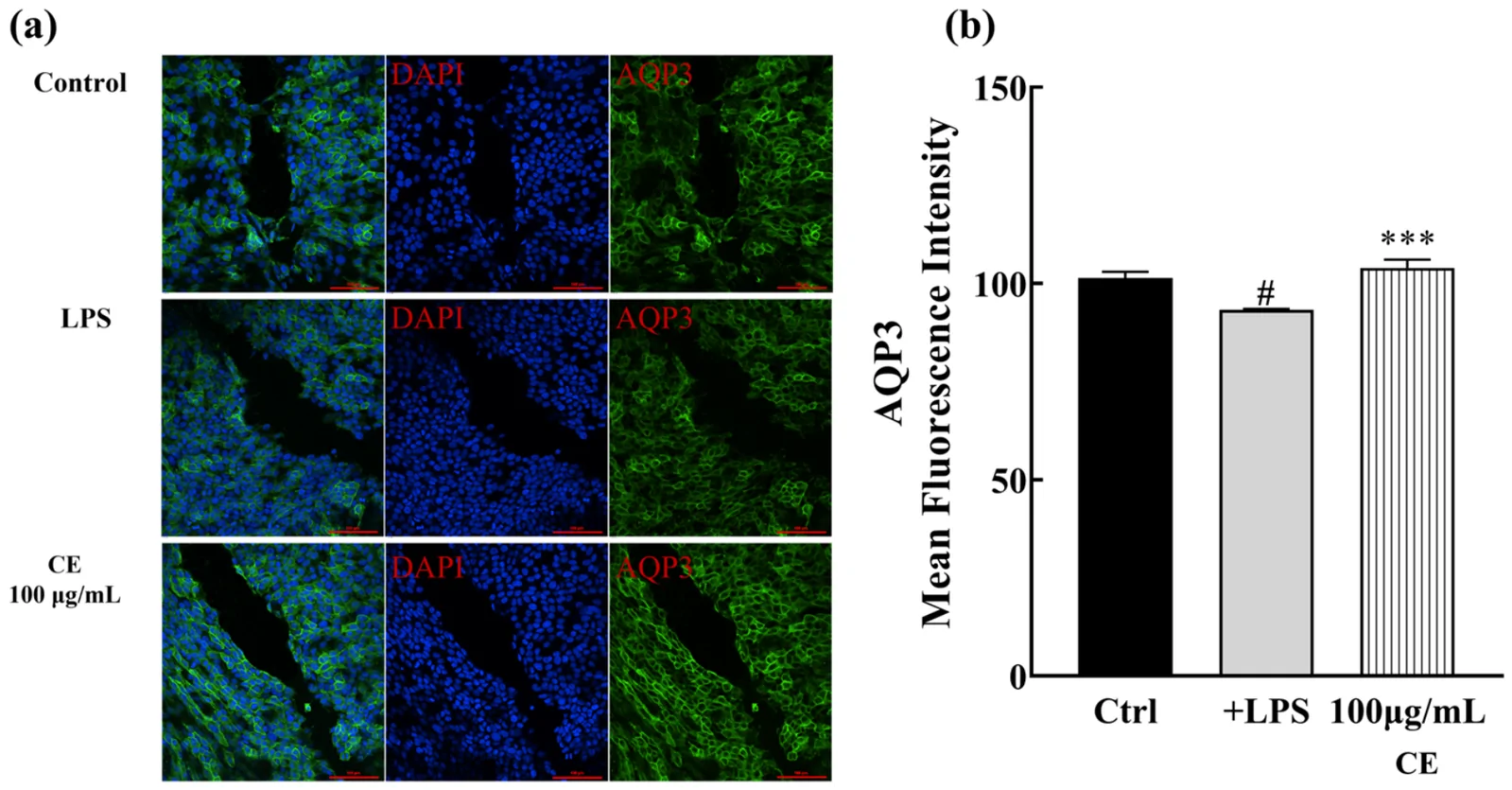
Effect of the CE on AQP3 expression in an in vitro wound healing model. (**a**) Fluorescence staining image showing the effect of the CE on AQP3 protein expression in HaCaT cells. Scale bar: 100 μm. (**b**) Quantitative analysis results of the fluorescence staining image showing the effect of CE on AQP3 protein expression in HaCaT cells. # *p* < 0.05 vs. C group; *** *p* < 0.001 vs. LPS group.

Immunofluorescence staining was used to quantitatively analyze the fluorescence intensity of aquaporin-3 (AQP3) and FLG in HaCaT cells at the scratch sites (Figure 8). The results revealed lower AQP3 fluorescence intensity at the scratch sites in the model group (scratch injury group) than in the blank control group. Following treatment with 100 μg/mL CE, the AQP3 fluorescence intensity increased significantly relative to that of the model group (*p* < 0.001), with a relative promotion rate of 11.50%.

**Figure 9:**
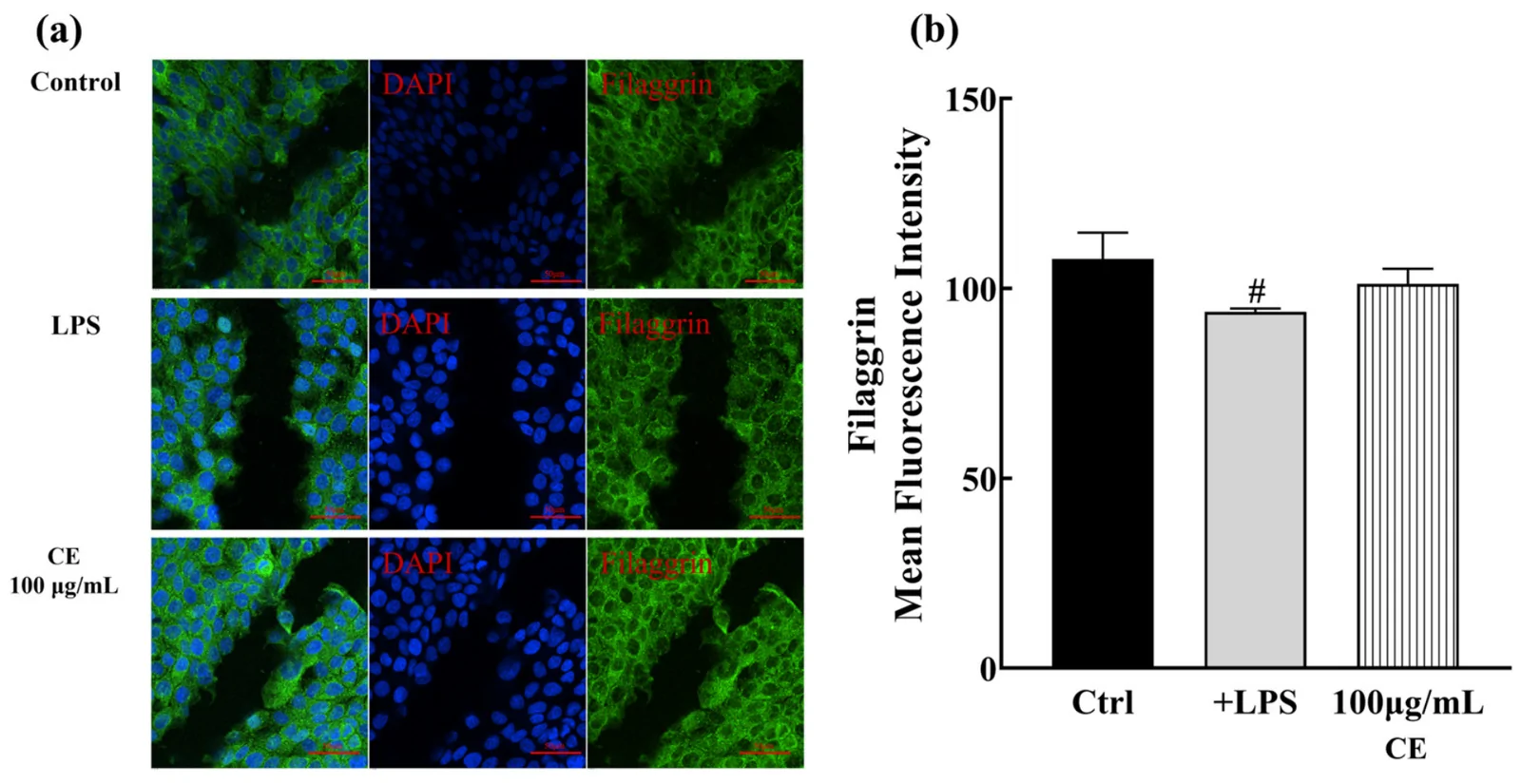
Effect of the CE on filaggrin (FLG) expression in an in vitro wound healing model. (**a**) Fluorescence staining image showing the effect of the CE on FLG protein expression in HaCaT cells. Scale bar: 50 μm. (**b**) Quantitative analysis results of the fluorescence staining image showing the effect of the CE on FLG protein expression in HaCaT cells. # *p* < 0.05 vs. C group.

Compared with the blank control group, the model group demonstrated reduced FLG fluorescence intensity at the scratch sites. Compared with the model group, the group treated with 100 μg/mL CE demonstrated a non-significant increase in FLG fluorescence intensity, with a promotion rate of 7.91% (Figure 9).

### 3.5. Effects of the CE on the Nuclear Translocation of Nrf2 and NFAT1

**Figure 10:**
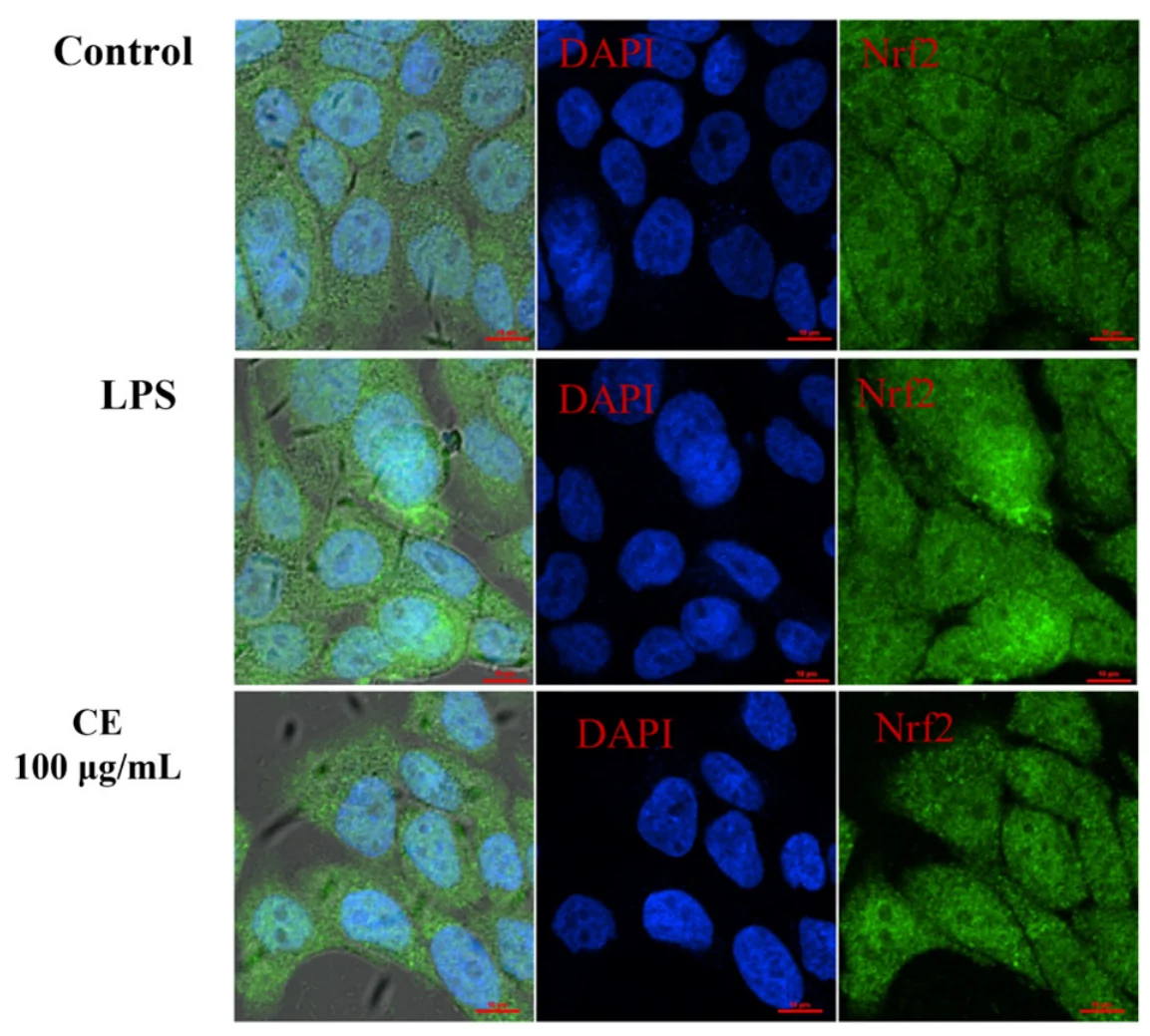
Effect of the CE on Nrf2 nuclear translocation in an in vitro wound healing model. Scale bar: 10 μm.

Immunofluorescence staining was used to examine the subcellular localization of Nrf2 in HaCaT cells in a model of non-chronic wound remodeling. The experimental design included the following three groups: blank control group (untreated), model group (no drug administered after wound creation), and treatment group (100 μg/mL CE administered concurrently with wound creation). In the blank control group, Nrf2 was primarily localized in the cytoplasm. In the model group, cells at the scratch site exhibited marked nuclear translocation of Nrf2, with increased nuclear fluorescence intensity relative to that of the blank control group (Figure 10). Compared with the model group, the treatment group exhibited reduced nuclear fluorescence intensity of Nrf2 and attenuated nuclear translocation.

**Figure 11:**
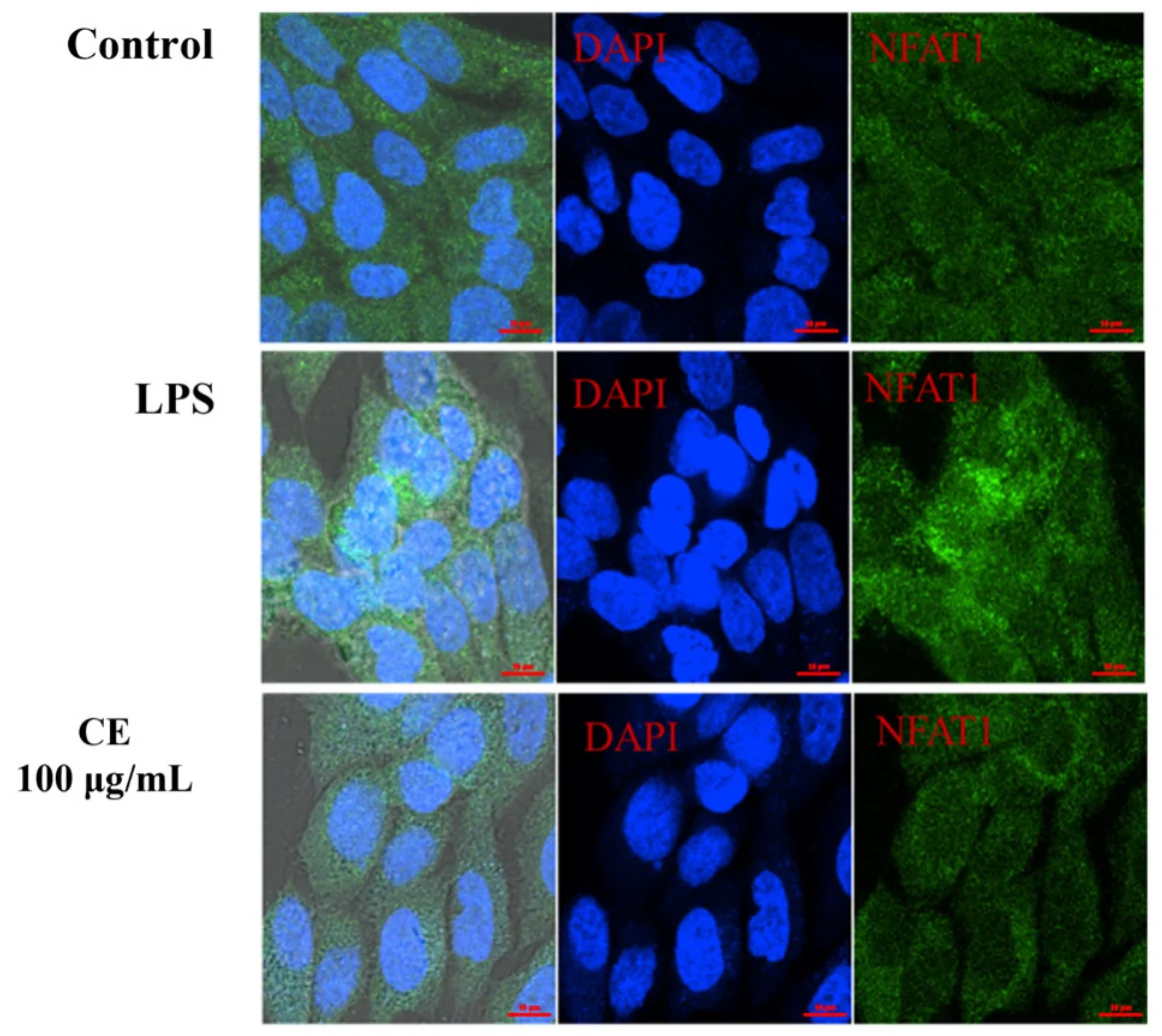
Effect of the CE on NFAT1 nuclear translocation in an in vitro wound healing model. Scale bar: 10 μm.

Immunofluorescence staining was used to examine the subcellular localization of NFAT1 in HaCaT cells in the different groups. In the blank control group, NFAT1 was primarily localized in the cytoplasm. In the model group, cells at the scratch sites exhibited marked NFAT1 nuclear translocation, with increased nuclear fluorescence intensity relative to that of the blank control group. Compared with the model group, the drug-treated group showed reduced NFAT1 nuclear fluorescence intensity and diminished nuclear translocation (Figure 11).

### 3.6. Effects of the CE on TGF-β1 Secretion in a Cell Model of Non-Chronic Wound Remodeling

**Figure 12:**
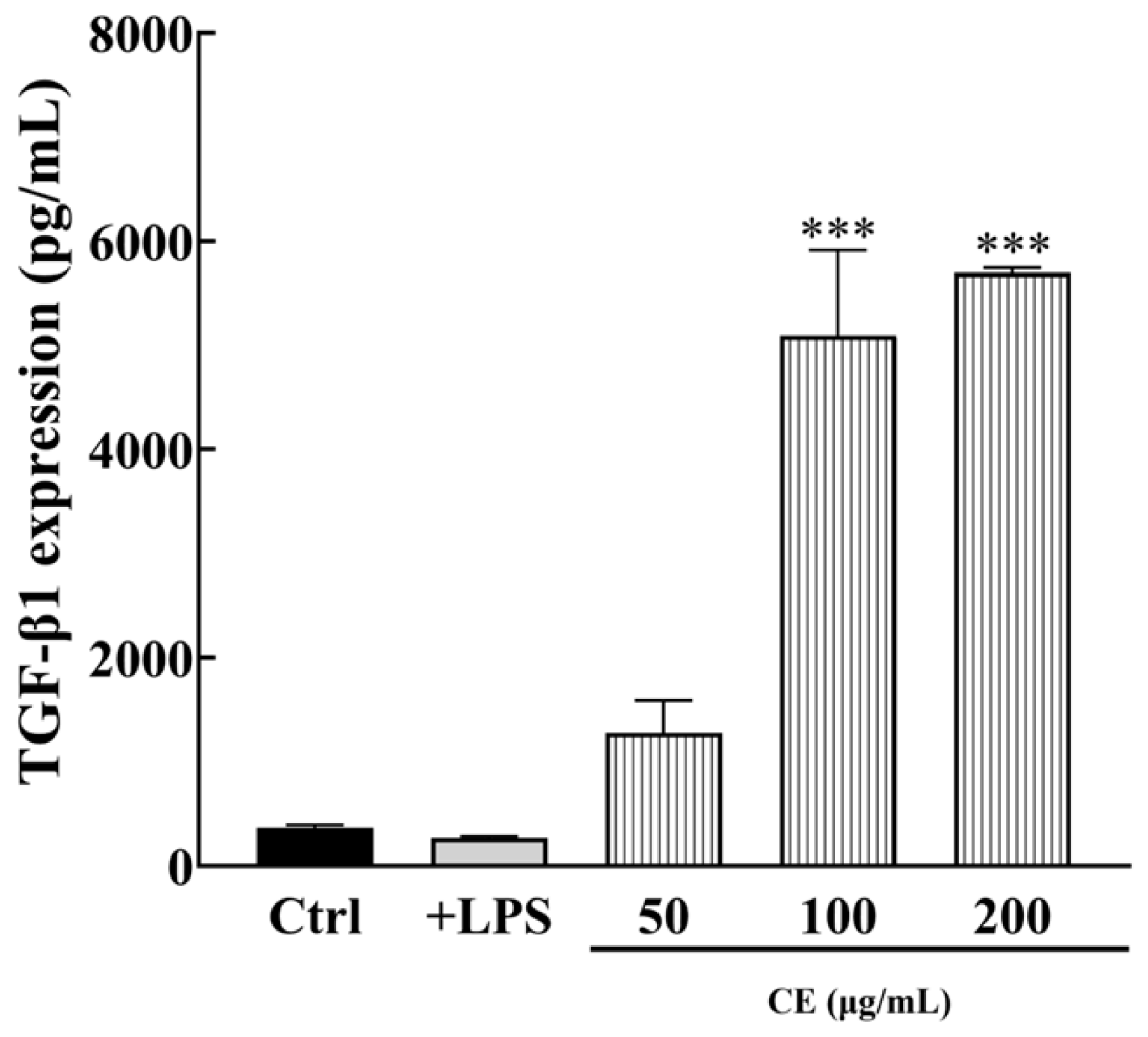
Effect of the CE on TGF-β1 expression in an in vitro wound healing model. *** *p* < 0.001 vs. LPS group.

TGF-β1 secreted by HaCaT cells, as a cell model of non-chronic wound remodeling, was quantified using enzyme-linked immunosorbent assay (ELISA). The experimental design included a blank control group (untreated), model group (no drug administered after wound creation), and treatment groups with varying concentrations of the CE (50, 100, and 200 μg/mL) administered concurrently with wound creation. Compared with the blank control group, the model group exhibited slightly reduced TGF-β1 secretion. Compared with the model group, the 100 and 200 μg/mL CE treatment groups exhibited significantly increased TGF-β1 secretion (*p* < 0.001); TGF-β1 secretion in the 200 μg/mL treatment group was 5694.78 pg/mL, which was a greater increase than that observed in the 100 μg/mL treatment group, demonstrating a concentration-dependent trend. The 50 μg/mL treatment group exhibited TGF-β1 secretion levels did not reach statistical significance relative to the model group (Figure 12).

### 3.7. Effects of the CE on the AKT/mTOR Signaling Pathway

**Figure 13:**
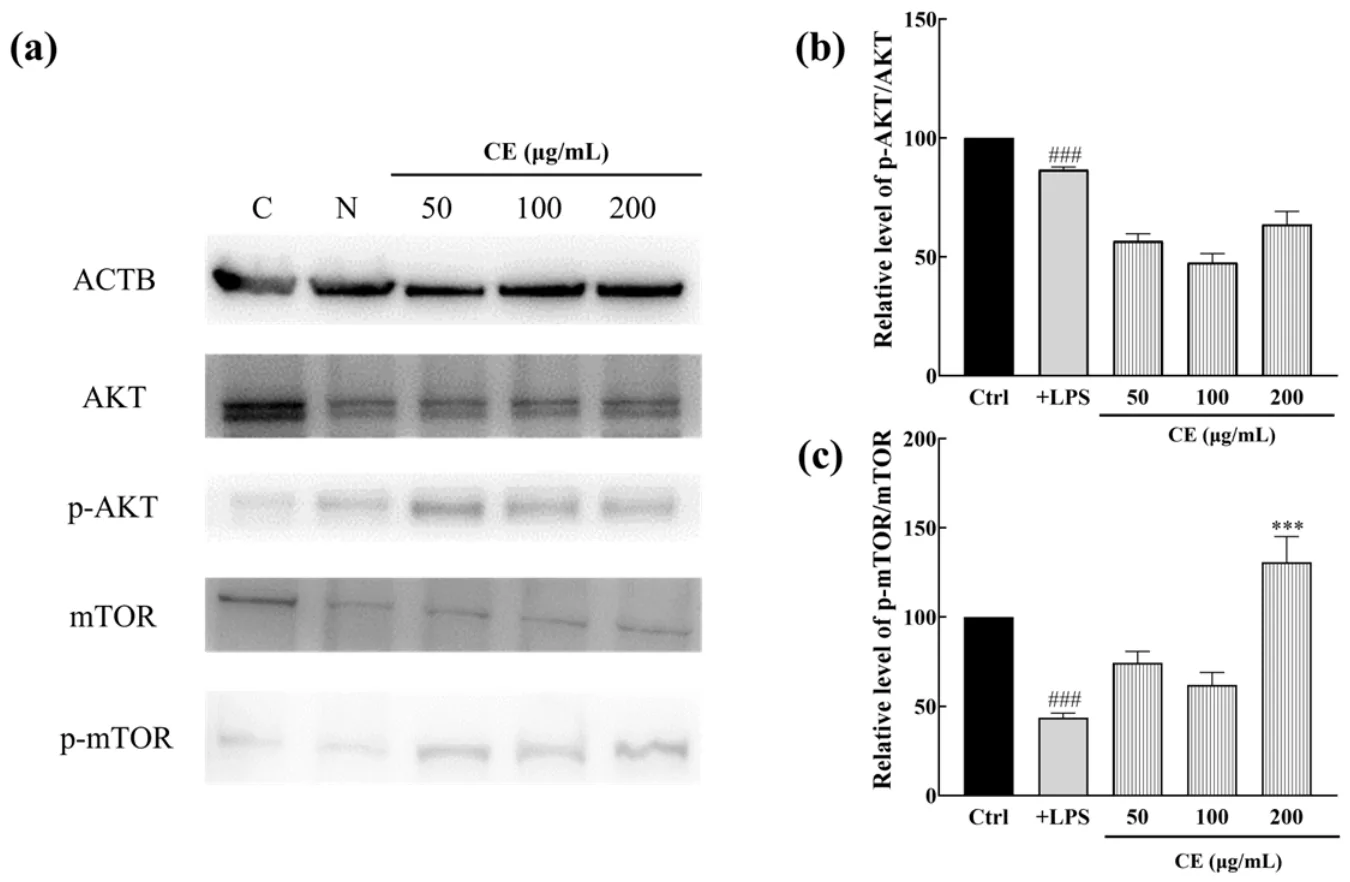
Effect of the CE on AKT/mTOR signaling in an in vitro wound healing model. (**a**) Western blot image showing the effect of the CE on AKT/mTOR protein expression in HaCaT cells. (**b**) Quantitative Western blot analysis results showing the effect of the CE on p-AKT/AKT protein expression in HaCaT cells. (**c**) Quantitative Western blot analysis results showing the effect of the CE on p-mTOR/mTOR protein expression in HaCaT cells. ### *p* < 0.001 vs. C group; *** *p* < 0.001 vs. N group.

Western blot analysis was used to detect the AKT and mTOR phosphorylation levels in HaCaT cells in the scratch assay model. The experimental design included a blank control group (untreated), model group (no drug added after model induction), and groups treated with varying CE concentrations (50, 100, and 200 μg/mL) simultaneously with model induction. Compared with the blank control group, the model group exhibited reduced p-AKT/AKT and p-mTOR/mTOR ratios. Neither the p-AKT/AKT ratio nor the p-mTOR/mTOR ratio showed a significant trend in the 50 and 100 μg/mL treatment groups, compared with those in the model group. The p-AKT/AKT ratio in the group treated with 200 μg/mL CE was not significantly different from that in the model group (*p* > 0.05), while the p-mTOR/mTOR ratio was significantly higher than that in the model group (*p* < 0.001) (Figure 13).

### 3.8. Effects of the CE on Erythrocyte Hemolysis

**Figure 14:**
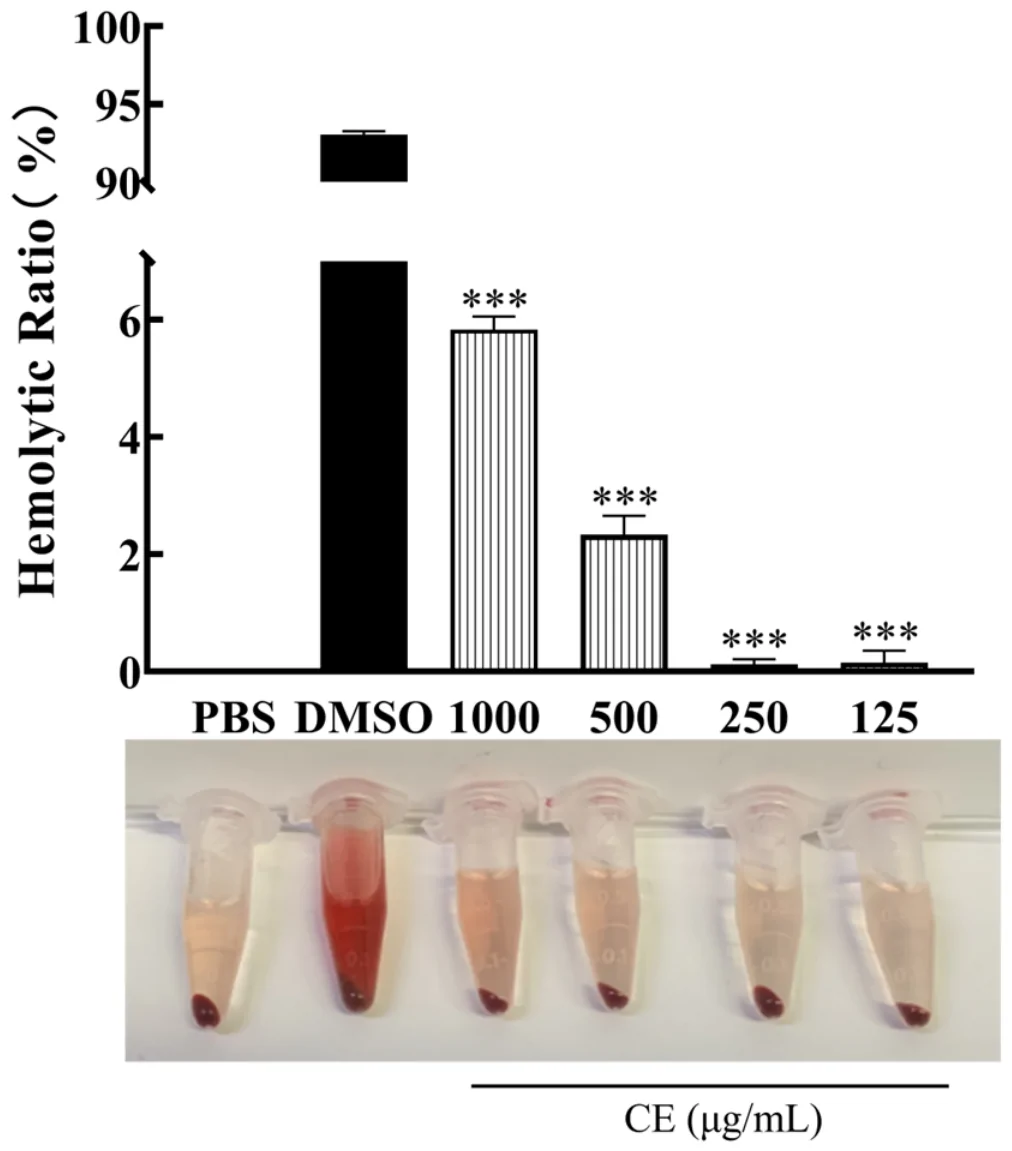
Effect of the CE on red blood cell hemolysis. *** *p* < 0.001 vs. DMSO group.

A hemolysis assay was used to evaluate the membrane-disrupting effects of the CE on red blood cells. The hemolysis rate was measured under 1.0 mg/mL CE condition, using phosphate-buffered saline (PBS) as the negative control and dimethyl sulfoxide (DMSO) as the positive control. The hemolysis rate of the positive control was 93.05%, while that of the negative control was close to 0%. At 1.0 mg/mL, the hemolysis rate of the CE was 5.83%, which was significantly lower than that of the positive control (Figure 14).

### 3.9. Effects of the CE on the Chicked Chorioallantoic Membrane (CAM)

**Figure 15:**
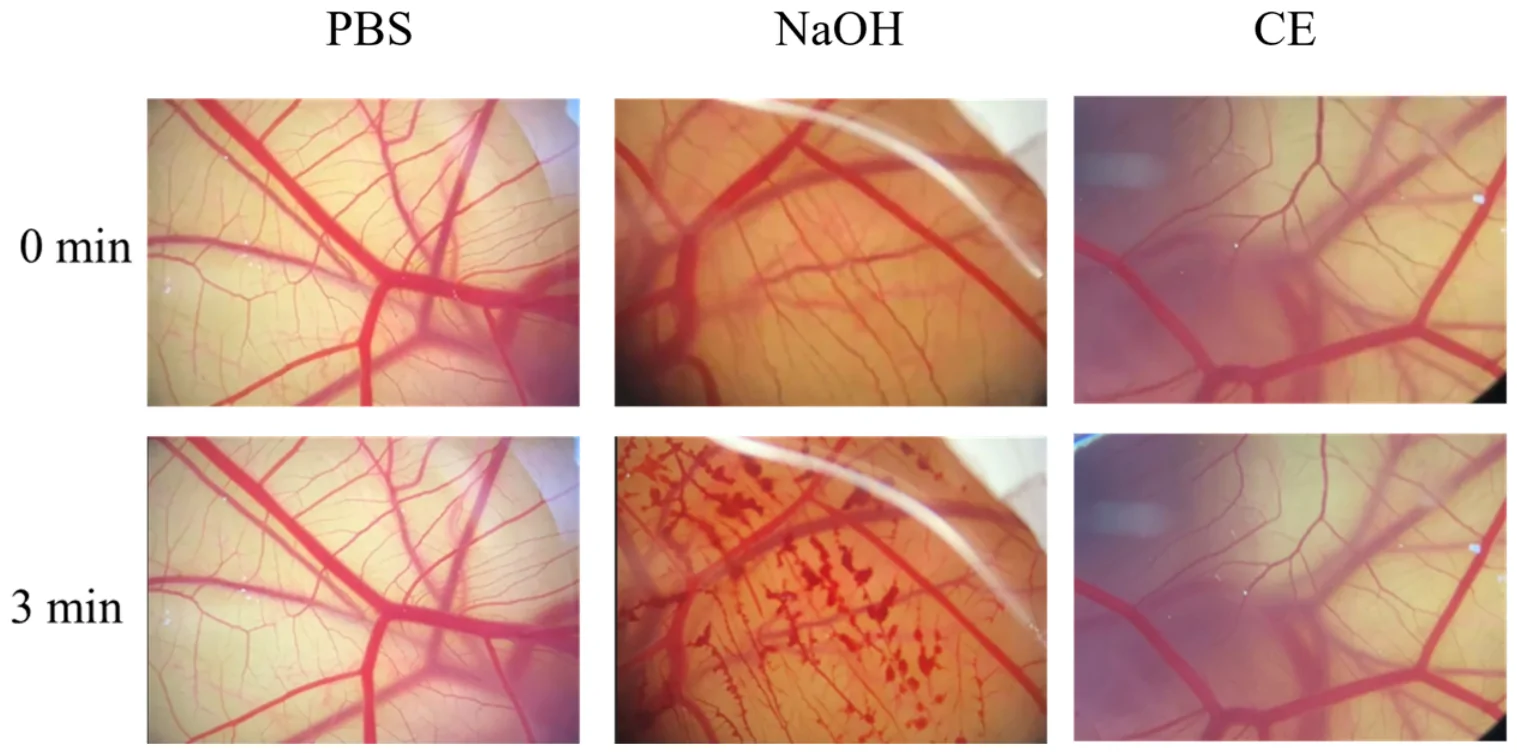
Effect of the CE on irritation of the chicken chorioallantoic membrane (CAM).

The chicken CAM assay was used to evaluate the vascular irritancy of the CE. The experimental design included a blank group (untreated), positive control group (0.1 mol/L NaOH addition), and treatment group (3.0 mg/mL CE addition). Vascular responses were continuously monitored following addition. The positive control group exhibited marked hemorrhage as early as 30 s after addition. The morphology of the CAM blood vessels in the treatment group was consistent with that in the control group, and no hemorrhage was observed (Figure 15).

## 4. Discussion

This study systematically evaluated the wound-healing potential of a CE from the following three perspectives: structural characteristics, cellular effects, and signaling responses. UV and IR spectral analyses were employed to characterize the extract. UV analysis indicated low levels of residual proteins and nucleic acids, suggesting that the extraction process effectively removed most of these macromolecular impurities. IR analysis confirmed the polysaccharide nature of the extract. The broad O–H stretching band in the 3200–3400 cm^−1^ region, red-shifted from the free O–H position (~3400 cm^−1^), indicates strong inter- or intra-molecular hydrogen bonding, consistent with previous reports on polysaccharide-rich extracts [34]. It should be noted that the original ATR-FTIR spectrum shows an anomalous transmittance (>100%T) at 3262.93 cm^−1^, which is a well-known artifact caused by residual moisture on the ATR crystal surface during background collection. Despite this artifact, the presence of O–H groups is supported by the carboxylate and C–O–C/C–O–H bands described below. The COO^−^ bands at 1638 and 1399 cm^−1^ (asymmetric and symmetric stretching) indicate the presence of uronic acid residues, while the strong band at 1050 cm^−1^ (C–O–C and C–O–H stretching), together with the band at 1219 cm^−1^ (C–O–H stretching), is characteristic of a furanose ring skeleton. These spectral features collectively confirm that CE is a polysaccharide extract containing uronic acid residues and a furanose backbone, with only trace amounts of residual proteins and nucleic acids. However, IR spectroscopy provides information only on functional groups and glycosidic linkage types; it cannot resolve the extract into individual polysaccharide constituents or identify which components are responsible for the observed bioactivities.

The GPC-based PDI of 6.65 suggested that the extract was a highly heterogeneous crude polysaccharide system. Consequently, the phytochemical composition of CE could not be resolved into individual constituents in this study, and no direct correlation between specific phytochemicals and the observed bioactivities could be established. The biological effects observed here should therefore be attributed to the “CE” as a composite system, rather than to any specific, homogeneous polysaccharide component [35,36]. Establishing such structure–activity relationships will require activity-guided fractionation and subsequent structural characterization.

At the cellular level, the extract did not exhibit significant cytotoxicity toward HaCaT cells within the range of 100–400 μg/mL, and a concentration of 100 μg/mL significantly promoted wound healing, indicating good in vitro biocompatibility and migration-promoting activity. Quantitative immunofluorescence results revealed that the scratch injury downregulated AQP3 expression, whereas polysaccharide treatment significantly ameliorated this change. Considering the important role of AQP3 in keratinocyte hydration, glycerol transport, and directed migration, this change may facilitate the re-epithelialization process [37,38]. In contrast, FLG expression increased but not significantly, suggesting that under the current experimental conditions, the repair-promoting effects of the extract are directed toward migration and hydration regulation rather than toward reconstructiion of barrier proteins associated with terminal differentiation. Furthermore, as the scratch healing process is influenced by both cell migration and proliferation, the relative contributions of these two factors in functional experiments need to be distinguished further through proliferation inhibition assays or real-time dynamic imaging [39].

At the signaling level, the nuclear translocation of Nrf2 and NFAT1 was enhanced in the model group, suggesting that the scratch injury simultaneously activated oxidative stress- and inflammation-related signaling pathways. Polysaccharide treatment reduced the nuclear localization of both these proteins. The nuclear translocation of Nrf2 represents a protective cellular response to oxidative damage; its reduced nuclear localization potentially reflects reduced upstream oxidative stress, and it does not provide direct evidence of antioxidant activity of this polysaccharide [40]. Follow-up studies should combine measurements of intracellular reactive oxygen species (ROS) levels, lipid peroxidation product levels, and expression of antioxidant target genes, such as heme oxygenase-1 (*HO-1*) and NAD(P)H quinone oxidoreductase 1 (*NQO1*), to further validate the ability of this extract to regulate oxidative stress. Furthermore, the suppression of NFAT1 nuclear translocation suggests that this polysaccharide may interfere with Ca^2+^/calmodulin-mediated inflammatory signaling pathways; however, causality remains to be confirmed via experiments involving calcium signaling interference or phosphatase inhibition [41,42].

ELISA findings revealed that the CE promoted TGF-β1 secretion in a concentration-dependent manner, and western blot analysis indicated that treatment with 200 μg/mL extract restored mTOR phosphorylation levels, while no significant changes were observed in AKT phosphorylation. These findings are consistent with the migration-promoting and repair-inducing phenotypes of the extract [43]; however, the current data are insufficient to establish TGF-β1 or mTOR as essential mediators of the repair effects. The absence of a concomitant increase in AKT phosphorylation suggests that mTOR activation may be mediated by a non-classical upstream pathway [44]. Future studies should establish a more comprehensive functional relationship within the signaling pathway by examining key nodes such as AMP-activated protein kinase (AMPK), regulated in development and DNA damage responses 1 and 2 (REDD1/2), p70S6K, and eukaryotic translation initiation factor 4E (eIF4E)-binding protein 1 (4E-BP1), in combination with mTOR-specific inhibitors [45]. Regarding safety evaluation, both the hemolysis and CAM assays provisionally indicated the biocompatibility of this extract [46,47].

Several limitations should nevertheless be acknowledged. First, the evidence was obtained exclusively from an in vitro HaCaT cell model, which cannot fully recapitulate the complex cellular, extracellular, vascular, and inflammatory microenvironment of cutaneous wound healing in vivo. In addition, the scratch-wound assay does not permit precise discrimination between the respective contributions of cell migration and proliferation to wound closure; future studies incorporating proliferation-inhibition controls, such as mitomycin C pretreatment, together with migration- and proliferation-specific assays, would help resolve this issue. Second, and central to the interpretation of the present results, the CE used in this study including crude polysaccharide mixtures with complex compositions. Although the spectral data confirm its polysaccharide nature, they do not identify which constituents are responsible for the observed activities. Thus, the key active components and their structure–activity relationships remain to be elucidated. Future studies should employ activity-guided fractionation and component-identification strategies to characterize the principal bioactive constituents, and should establish appropriate animal wound-healing models to evaluate the in vivo efficacy, local safety, tissue-level responses, and translational potential of Chlorella-derived extracts. Furthermore, pharmacological inhibition and genetic intervention approaches will be valuable for clarifying the causal relationships among AQP3-mediated migration, oxidative stress, inflammatory responses, and mTOR-related signaling. Collectively, these investigations will provide a more robust scientific basis for the development of safe and effective CE wound-healing formulations.

## 5. Conclusions

The CE exhibited promising wound-healing potential in vitro; its effects may be associated with AQP3-mediated migration and hydration regulation, as well as with the modulation of oxidative stress-, inflammatory-, and mTOR-related signaling pathways. However, the present study did not establish a direct correlation between specific phytochemical constituents and the observed activities. Given that CE is a complex crude polysaccharide mixture, its bioactivity likely arises from the combined or synergistic effects of multiple components rather than from a single compound. Therefore, the phytochemical composition and the measured activities can only be tentatively linked at this stage, and such associations remain hypothetical rather than causal.

Considering that the current evidence is primarily derived from an in vitro HaCaT cell model and that the samples consist of complex crude polysaccharide mixtures, future studies should combine activity-guided isolation with structural characterization to identify the key active constituents and clarify their structure–activity relationships. Such approaches, together with animal wound models and signaling pathway inhibition experiments, will provide a more robust experimental basis for correlating specific phytochemicals with their wound-healing activities and for developing CE-based wound-healing formulations.

## Figures and Tables

**Table 1:** Assignment of FTIR absorption peaks for the CE.

Absorption Peak (cm^−1^)	Corresponding Structure/Vibration
3200–3400	O–H stretching vibration (hydrogen-bonded, broad peak)
2975	C–H stretching vibration (–CH and –CH_2_ groups of carbohydrates)
1638	Asymmetric stretching vibration of COO^−^
1399	symmetric stretching of COO^−^ from uronic acids; may overlap with C–O–H bending
1219	C–O stretching (alcoholic/phenolic groups)
1050	C–O stretching vibration (C–O–H/C–O–C in furanose ring)

## Data Availability

The raw data supporting the conclusions of this article will be made available by the authors on request.

## Abbreviations

The following abbreviations are used in this manuscript:

AKT	Protein kinase B
AQP3	Aquaporin-3
CAM	Chicken chorioallantoic membrane
CCK-8	Cell Counting Kit-8
CE	*Chlorella vulgaris* extract
DMEM	Dulbecco’s Modified Eagle Medium
DMSO	Dimethyl sulfoxide
ECL	Enhanced chemiluminescence
ECM	Extracellular matrix
ELISA	Enzyme-linked immunosorbent assay
EMT	Epithelial–mesenchymal transition
4E-BP1	Eukaryotic translation initiation factor 4E (eIF4E)-binding protein 1
FLG	Filaggrin
FTIR	Fourier-transform infrared spectroscopy
GPC	Gel permeation chromatography
HO-1	Heme oxygenase-1
IL	Interleukin
IR	Infrared
LPS	Lipopolysaccharide
mTOR	Mechanistic target of rapamycin
NFAT	Nuclear factor of activated T-cells
Nrf2	Nuclear factor erythroid 2-related factor 2
NQO1	NAD(P)H quinone oxidoreductase 1
PBS	Phosphate-buffered saline
PI3K	Phosphatidylinositol 3-kinase
REDD1/2	Regulated in development and DNA damage responses 1 and 2
ROS	Reactive oxygen species
TGF-β1	Transforming growth factor-β1
